# Task-Switching Performance Improvements After Tai Chi Chuan Training Are Associated With Greater Prefrontal Activation in Older Adults

**DOI:** 10.3389/fnagi.2018.00280

**Published:** 2018-09-24

**Authors:** Meng-Tien Wu, Pei-Fang Tang, Joshua O. S. Goh, Tai-Li Chou, Yu-Kai Chang, Yung-Chin Hsu, Yu-Jen Chen, Nai-Chi Chen, Wen-Yih Isaac Tseng, Susan Shur-Fen Gau, Ming-Jang Chiu, Ching Lan

**Affiliations:** ^1^School and Graduate Institute of Physical Therapy, College of Medicine, National Taiwan University, Taipei, Taiwan; ^2^Yonghe Cardinal Tien Hospital, Taipei, Taiwan; ^3^Graduate Institute of Brain and Mind Sciences, College of Medicine, National Taiwan University, Taipei, Taiwan; ^4^Department of Physical Medicine and Rehabilitation, National Taiwan University Hospital, Taipei, Taiwan; ^5^Neurobiology and Cognitive Science Center, National Taiwan University, Taipei, Taiwan; ^6^Center for Artificial Intelligence and Robotics, National Taiwan University, Taipei, Taiwan; ^7^Department of Psychology, College of Science, National Taiwan University, Taipei, Taiwan; ^8^Department of Physical Education, National Taiwan Normal University, Taipei, Taiwan; ^9^Institute of Medical Device and Imaging, College of Medicine, National Taiwan University, Taipei, Taiwan; ^10^Department of Psychiatry, National Taiwan University Hospital, Taipei, Taiwan; ^11^Department of Neurology, National Taiwan University Hospital, Taipei, Taiwan

**Keywords:** aging, cognition, executive function, functional neuroimaging, Tai Chi Chuan, exercise intervention

## Abstract

Studies have shown that Tai Chi Chuan (TCC) training has benefits on task-switching ability. However, the neural correlates underlying the effects of TCC training on task-switching ability remain unclear. Using task-related functional magnetic resonance imaging (fMRI) with a numerical Stroop paradigm, we investigated changes of prefrontal brain activation and behavioral performance during task-switching before and after TCC training and examined the relationships between changes in brain activation and task-switching behavioral performance. Cognitively normal older adults were randomly assigned to either the TCC or control (CON) group. Over a 12-week period, the TCC group received three 60-min sessions of Yang-style TCC training weekly, whereas the CON group only received one telephone consultation biweekly and did not alter their life style. All participants underwent assessments of physical functions and neuropsychological functions of task-switching, and fMRI scans, before and after the intervention. Twenty-six (TCC, *N* = 16; CON, *N* = 10) participants completed the entire experimental procedure. We found significant group by time interaction effects on behavioral and brain activation measures. Specifically, the TCC group showed improved physical function, decreased errors on task-switching performance, and increased left superior frontal activation for Switch > Non-switch contrast from pre- to post-intervention, that were not seen in the CON group. Intriguingly, TCC participants with greater prefrontal activation increases in the switch condition from pre- to post-intervention presented greater reductions in task-switching errors. These findings suggest that TCC training could potentially provide benefits to some, although not all, older adults to enhance the function of their prefrontal activations during task-switching.

## Introduction

Task-switching allows a person to rapidly and flexibly adapt behaviors to respond to multi-task rules and demands (Braver et al., 2003) such as when dealing with dynamic changes in complex environments. This high-level executive function involves several sub-processes that include attention, classification, inhibition, updating, memory retrieval and response to stimulus (Monsell, 2003). Past studies have documented that task-switching ability consistently declines with age. Compared to young adults, older adults present significantly poorer accuracy and slower reaction time (RT) while performing task-switching tasks (Kray and Lindenberger, 2000; DiGirolamo et al., 2001; Reimers and Maylor, 2005; Wasylyshyn et al., 2011; Gazes et al., 2012; Zhu et al., 2014; Hakun et al., 2015). Aged-related task-switching declines are associated with functional mobility declines in daily living (Hawkes et al., 2012; Gothe et al., 2014; Blackwood et al., 2016). Thus, the prevention or alleviation of declines in task-switching ability in older adults is an important cognitive aging issue that motivates this present study.

Tai Chi Chuan (TCC) exercise is a form of traditional Chinese exercise that involves physical activity, cognitive control, as well as social interaction when practiced in a group. Individuals who practice TCC often gain not only improvements in physical function and psychological well-being (Taylor-Piliae, 2008; Wang et al., 2010; Lan et al., 2013; Chan et al., 2017), but also in several domains of cognitive function, including global cognitive function, attention, language, perception, learning, memory and in particular executive function (Taylor-Piliae et al., 2010; Wayne et al., 2014; Zheng et al., 2015). Indeed, the beneficial effects of TCC training on executive function in cognitively intact older adults have been consistently reported in Trail-Making-Test Part B (TMT-B) performance (Matthews and Williams, 2008; Mortimer et al., 2012; Nguyen and Kruse, 2012; Wayne et al., 2014). The TMT-B probes task-switching and working memory related abilities by requiring participants to process behavioral response rules that shift between two different stimulus categories (letter vs. number; Reitan, 1958; Sánchez-Cubillo et al., 2009). In a one group pretest-posttest design study, TMT-B performance of older adults was shown to improve after a 10-week TCC training intervention (Matthews and Williams, 2008). Moreover, TMT-B performance also significantly improved after 6 months of TCC training in randomly assigned community-dwelling older adults compared to routine-care controls (Nguyen and Kruse, 2012).

Evidence also suggests that TCC related interventions may be associated with specific changes of brain structure as well as resting-state connectivity and activation of the brain (Mortimer et al., 2012; Wei et al., 2013, 2014, 2017; Li et al., 2014; Yin et al., 2014). Cross-sectional studies revealed that TCC experts with an average of 14 years of experience, compared with non-TCC practitioners, had greater gray matter cortical thickness in right middle frontal, insula and precentral, and left superior temporal, medial occipito-temporal and lingual regions (Wei et al., 2013), and showed smaller functional homogeneity in the dorsolateral prefrontal cortex (DLPFC) and anterior cingulate cortex, with the latter findings suggesting enhanced functional specialization of brain regions involved in attention control (Wei et al., 2014). Also using resting-state functional magnetic resonance imaging (fMRI), Wei et al. (2017) found a lower fractional amplitude of low frequency fluctuations (fALFF) in the blood oxygen level dependent (BOLD) signal of bilateral frontoparietal attention network during rest in these experienced TCC practitioners, compared to non-practitioners, with lower fALFF correlating with better attention control performance assessed using Attention Network Test response time. While these cross-sectional studies may indicate that long-term TCC practice is associated with altered brain structure and resting-state functional specialization of certain brain regions, it remains difficult to establish causal relationships between TCC practice and these brain changes because of the limitations of the cross-sectional nature of the study design.

Several longitudinal studies using resting-state fMRI have provided more direct evidence in support of the effects of TCC-related practice on changing functional connectivity of the brain and the associated cognitive function after intervention (Li et al., 2014; Yin et al., 2014; Tao et al., 2017). Li et al. (2014) reported that after a 6-week multimodal intervention program comprising TCC training, cognitive training, and group counseling, older adults increased brain functional connectivity between the medial PFC and medial temporal lobe in their resting-state fMRI, and this increase of functional connectivity was associated with better task-switching and category fluency performance. Also using resting-state fMRI, Yin et al. (2014) found that after a similar 6-week multimodal training program, older adults enhanced the ALFF, the regional spontaneous activation during rest, in the middle frontal gyrus (MFG), superior frontal gyrus (SFG), and the cerebellum, and that greater increases of ALFF in the right MFG were associated with better TMT-B performance improvements. Tao et al. (2017) also found that older adults who received a 12-week TCC intervention program also increased fALFF in the DLPFC, and this increase was associated with improvement of their memory function. Note, reduced fALFF of the resting-state frontoparietal network was found in TCC experts in one cross-sectional study (Wei et al., 2017), whereas increased ALFF or fALFF in the resting-state prefrontal network was found in older adults who undertook short-term TCC intervention in two longitudinal studies (Yin et al., 2014; Tao et al., 2017). This discrepancy may stem from differences in the duration of TCC practice (long- vs. short-term), study design (cross-sectional vs. longitudinal), or brain regions used to calculate the functional connectivity indices. Nevertheless, long- and short-term TCC training both resulted in detectable differences in resting-state functional connectivity in older adult participants. Importantly, intervention studies that investigate the effects of TCC training on task-switching related functional brain processes using task-fMRI in older adults are still lacking (Yu et al., 2018). Thus, it remains unclear whether and how TCC training alters task-switching associated brain functional responses and how these changes in functional responses correlate with improvement in task-switching ability.

Previous studies that investigated task-switching associated brain functional activation found that compared to non-switch trials, task-switching trials evoked higher neural activity in the SFG and MFG, inferior frontal gyrus pars triangularis (IFG_t_) and opercularis (IFG_o_), medial PFC, and superior and inferior parietal cortices (DiGirolamo et al., 2001; Sakai and Passingham, 2003; Crone et al., 2006; Gold et al., 2010; Jimura and Braver, 2010; Zhu et al., 2014; Hakun et al., 2015). Among these frontoparietal regions, age differences are characterized with young adults predominately recruiting the left DLPFC and inferior frontal gyrus (Dove et al., 2000; MacDonald et al., 2000; Kim et al., 2011) and older adults recruiting bilateral prefrontal regions (DiGirolamo et al., 2001; Gazes et al., 2012; Zhu et al., 2014; Hakun et al., 2015). However, over-recruitment in prefrontal regions was negatively associated or unassociated with task-switching performance during Switch conditions in older adults, indicating an attempted but unsuccessful compensatory mechanism (Cabeza and Dennis, 2013; Zhu et al., 2014; Hakun et al., 2015). Noting the above beneficial effects of TCC practice on task-switching performance, we aimed to more specifically determine how TCC training alters prefrontal functional responses associated with task-switching in cognitively normal older adults and how these changes were associated with their task-switching performance.

We conducted a randomized controlled clinical trial (RCT) task-switching fMRI study to investigate changes in task-switching performance and associated brain functional activation, focusing on bilateral prefrontal regions, before and after a 12-week TCC intervention in cognitively intact older adults. Bilateral prefrontal regions were our foci because they are heavily engaged during task-switching and evinced age differences in prior studies (DiGirolamo et al., 2001; Sakai and Passingham, 2003; Crone et al., 2006; Gold et al., 2010; Jimura and Braver, 2010; Zhu et al., 2014; Hakun et al., 2015). In addition, functional activation in prefrontal regions often increases during executive function tasks after aerobic or resistance exercise interventions (Colcombe et al., 2004; Liu-Ambrose et al., 2012). We hypothesized that older adult who received TCC intervention would improve task-switching ability and increase task-switching associated prefrontal functional activation during task-switching fMRI and that such activity increases would positively correlate with post-intervention task-switching performance improvement.

Task-switching fMRI experimental paradigms typically involve measuring brain and behavioral responses during Non-switch and Switch conditions. In Non-switch conditions, participants respond to consecutive trials involving the same single task rule (e.g., AAAAAA or BBBBBB for six trials with rules A and B). In Switch conditions, participants alternate, sometimes stochastically, between a mix of two task rules (e.g., ABAABBAB). Behavioral performance in task-switching can be assessed via accuracy and RT of responses in Switch conditions (Kray and Lindenberger, 2000; Reimers and Maylor, 2005). Alternatively, a global Switch cost measure can also be derived that uses differences in error rates and RTs between averages across trials for Non-switch and Switch conditions (Switch-Non-switch condition values; Kray and Lindenberger, 2000; Wasylyshyn et al., 2011). Yet another approach is to compute a local Switch cost, which uses differences in error rates and RTs of consecutive Non-switch and Switch trials (Switch-Non-switch trial values) within the Switch condition (Monsell, 2003; Wasylyshyn et al., 2011). Past studies have shown that RT and accuracy in Switch conditions and global Switch cost in RTs are both sensitive to aging (Kray and Lindenberger, 2000; Reimers and Maylor, 2005; Wasylyshyn et al., 2011), whereas local Switch cost does not change with age (Wasylyshyn et al., 2011). Therefore, in this study, we focused on Switch condition and global Switch cost indices of the task-switching fMRI experiment to evaluate the effects of TCC training on task-switching behavioral and neural responses.

In addition to using task-switching behavioral measures in the fMRI experiment, we also included the Intra/Extra-Dimensional Set Shift (IED) test of the Cambridge Neuropsychological Test Automated Battery (CANTAB; Cambridge Cognition Ltd., Bottisham, Cambridge, UK) to assess whether the effects of TCC training could be detected by a clinical assessment of the construct of task-switching (Robbins et al., 1998) that was independent from our fMRI task-switching experiment. Importantly, the IED test has established construct validity (Kim et al., 2014), good test-retest reliability (Henry and Bettenay, 2010), and has been used to detect cognitive declines in aging (Robbins et al., 1998) or prefrontal/fronto-striatal dysfunction (Fray et al., 1996). IED use was also in consideration of potential ceiling effects on inside-fMRI task-switching performance due to the practice criterion set in our fMRI experiment (described in “fMRI Number Stroop Task and Procedure” section). Overall, we hypothesized that performance on the IED test would be improved after a 12-week intervention in the TCC group only and that this improvement would be associated with increases in prefrontal activation during task-switching from pre- to post-intervention.

## Materials and Methods

### Participants and Study Design

**Figure 1:**
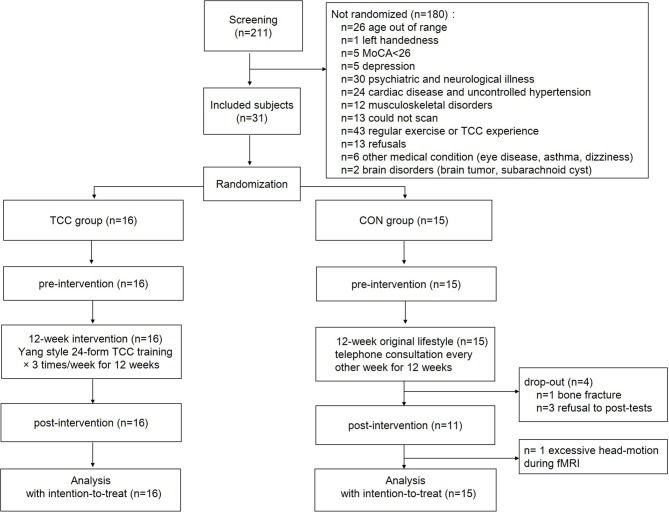
Consort chart of the randomized controlled trial.

This was an assessor-blind RCT (Figure 1). Participants (*N* = 31; age range 60–69 years old) were chosen from a larger sample of participants who were enrolled in a registered RCT study^1^ and were randomly assigned to either the TCC or the control (CON) group using the stratified randomization method based on age range. The study protocol was registered at ClinicalTrials.gov (NCT02270320). All participants underwent neuropsychological and physical function assessments, as well as structural and functional brain MRI scans before and after the 12-week intervention period. This study was carried out in accordance with the recommendations of “Research Ethics Committee, National Taiwan University Hospital” with written informed consent from all subjects. All subjects gave written informed consent in accordance with the Declaration of Helsinki. The protocol was approved by the “Institutional Review Board, National Taiwan University Hospital (No. 201212161RIND)”.

Participants were recruited from local communities in the Taipei metropolitan area. Inclusion criteria were aged between 60–69 years, education level ≥6 years, right-handedness (Oldfield, 1971), right-footedness (Elias et al., 1998) and native Mandarin speakers. Exclusion criteria were Montreal Cognitive Assessment Taiwan version (MoCA) score <26 (Nasreddine et al., 2005; Tsai et al., 2012), Clinical Dementia Rating score >0 (Hughes et al., 1982), Geriatric Depression Scale 15-item short-form (GDS-15) score >8 (Nyunt et al., 2009), Instrumental Activities of Daily Living disability items ≥1 (Lawton and Brody, 1969), psychiatric and neurological illness, severe or uncontrolled cardiovascular diseases or musculoskeletal disorders, any MRI contraindications, regular moderate-intensity exercise habits (defined as >30 min per session and more than three sessions per week in the past 6 months), and prior experiences with TCC, yoga, qigong, or martial arts practice. To further understand the required sample size of participants, we calculated the sample size by using the G*Power 3.1.9.2 software (Faul et al., 2009). In this calculation, we assumed a moderate effect (effect size of Hedge’s g = 0.51) of TCC training on executive function (Wayne et al., 2014) with the α level set at 0.05 and beta set at 0.2, using in a 2 (group) by 2 (time point) two-way repeated measures analysis of variance (RM ANOVA) design. The results showed that to reach a power of 0.8, the required total sample size was 26. Assuming a 20% dropout rate, the final total sample size of 31 was determined.

### Tai Chi Chuan Intervention and Control Procedures

The TCC group received three weekly sessions of 24-form Yang-style TCC (Liang and Wu, 1996) group training for 12 weeks. A 12-week intervention period was chosen because previous behavioral and resting-state fMRI studies suggested that a 12-week TCC program is sufficient to show cognitive effects and alter resting-state brain activation and connectivity in older adults (Wayne et al., 2014; Tao et al., 2016, 2017) and because the duration provided sufficient time for our older participants to steadily learn 2–3 new forms of the 24-form Yang-style TCC weekly. Each training session, consisting of 10 min of warm-up, 10 min of new TCC form learning, 30 min of continuous sequential practice of learned forms, and 10 min of cool-down, was led by a certified TCC coach with more than 10 years of TCC coaching experiences. Using the Polar Watch (Polar Electro Oy, Kempele, Finland) to monitor participants’ heart rate during the exercise sessions, we found that the intensity of the 30-min continuous TCC practice reached approximately 65.4 ± 1.1% (range = 63.8% to 66.7%) of individual participant’s age-predicted maximal heart rate (HR_max_) on average, and thus could be considered moderate intensity (64% to 76% HR_max_) endurance exercise according to the classification of American College of Sports Medicine (Lan et al., 2008; Garber et al., 2011). The CON group was instructed to maintain their original daily routines and physical activity habits and not to receive any new or additional exercise interventions. All CON participants received one telephone consultation biweekly during the 12-week period and their physical activity level and frequency of social interaction were recorded using the Physical Activity Scale for the Elderly (PASE; Washburn et al., 1993). Free TCC training course was offered to them after the study period.

### Neuropsychological and Physical Function Assessments

Before and after the intervention period, we measured participants’ task-switching behavioral performances by using the IED test, as well as the outside- and inside-fMRI task-switching behavioral performance measures (described in “fMRI Number Stroop Task and Procedure” section). The IED test has a maximum of nine stages of increasing difficulty, ranging from simple stimulus-response discrimination, reversal learning, compound discrimination, intra-dimensional set-shifts, to difficult extra-dimensional set-shifts. Having received no explicit instructions on when the intra- or extra-dimensional trials began or ended, participants had to figure out whether the intra- vs. extra-dimensional rules have changed in each trial according to the “correct” or “wrong” feedback displayed from the computer screen to his/her response to the previous trial. In particular, in the stage of intra-dimensional set-shift trials, participants had to selectively maintain attention on the same specific dimension (such as form) of the stimuli across trials, whereas in the stage of extra-dimensional shift trials, they had to switch attention to a previously irrelevant stimulus dimension (such as line; Fray et al., 1996; Robbins et al., 1998). Each stage has a maximum of 50 trials and could be ended prematurely when the participant correctly answered six trials of that stage consecutively. In this case, without notification, the subject would be prompted to the next stage, in which a different rule is used. The entire IED would be terminated at the end of the stage in which the subject could not correctly answer six trials consecutively of that stage. We used the number of completed stages and the number of total errors of all answered trials on the IED test in this study, with a higher number of completed stages or a smaller number of total errors indicating better task-switching performance.

To allow for understanding the associations among changes in physical function, cognitive function, and brain activation after TCC training, we also conducted pre- and post-intervention physical function tests of muscle strength, balance, mobility, and cardiorespiratory endurance. The muscle strength of bilateral knee extensors was measured twice with a handheld dynamometer (Lafayette Instrument Co., Lafayette, IN, USA; Wang et al., 2002). The better performance of the two trials was recorded for each leg and then averaged to represent the strength. Balance ability was assessed with the eyes-open one-legged stance test (OLST) up to 30 s for each of five trials (Bohannon et al., 1984). The best trial performance of the dominant leg was recorded. Mobility was assessed with two trials each of the Four Square Step Test (FSST; Dite and Temple, 2002). The better performance of the two trials was recorded. Cardiorespiratory endurance was assessed with one trial of the 6-Minute Walk Test (6MWT; Brooks et al., 2003). We also recorded each participant’s physical activity level using the PASE before, during, and after the intervention (Washburn et al., 1993). Participants’ frequency of being engaged in social interaction activities was extracted from narratives of their answers to questions 1 and 6 of the PASE.

### fMRI Number Stroop Task and Procedure

**Figure 2:**
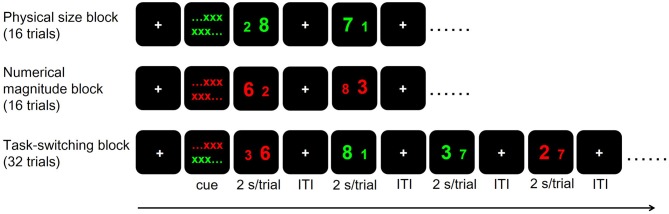
The hybrid block/event-related task-switching functional magnetic resonance imaging (fMRI) paradigm. Each trial lasted 2 s and the inter-trial interval (ITI) varied among 2, 4 and 6 s. Cue: color-cued instructions, with green indicating physical size rule and red indicating numerical magnitude rule.

We adopted a hybrid block/event-related task-switching fMRI paradigm modified from Huang et al. (2012). The paradigm was implemented using E-prime 2.0 (Psychology Software Tools, Pittsburgh, PA, USA). For each participant, we applied two runs of fMRI scans (7 min/run) each in the before- and after-intervention time points. Each run included two Non-switch blocks (physical size and numerical magnitude blocks in counter-balanced order across subjects, 16 trials for each block) first, followed by a Switch block (32 trials; Figure 2). In each trial, participants were presented with a pair of digits and were required to distinguish the digits by following the cues given before each block. In the physical size block, participants distinguished which digit of the two (with Arial font sizes of 73 and 55) were physically larger than the other, ignoring their numerical magnitude. In the numerical magnitude block, they distinguished which of the two digits (that differed by 3, 4, or 5) was numerically larger than the other, ignoring their physical size. Digit pairs were colored green and red in physical size and numerical magnitude blocks, respectively. In the Switch block, participants had to distinguish the physical size or numerical magnitude of the two digits according to stimuli color, with green indicating physical size distinction and red indicating numerical magnitude distinction. Each trial lasted 2 s, with pseudo-randomly jittered fixation inter-trial-intervals ranging between 2, 4 and 6 s. Each block was preceded by a 20-s fixation resting duration followed by 2 s of color-cued instructions on the relevant task dimension(s) for that block. Note that physical size and numerical magnitude of the two digits could be congruent or incongruent in a given trial. There were an equal number of congruent and incongruent trials for physical size, numerical magnitude and Switch blocks in both runs. Before entering the scanner, all participants received two short-practice sessions with 40 trials each (10 physical size trials, 10 numerical magnitude trials and 20 Switch trials) and reached ≥70% accuracy. The error rates (in percentage) during the practice sessions were used to compute the outside-fMRI Non-switch, Switch and Switch cost behavioral performances, and the error rates and the mean RT collected during the fMRI scans were used to compute the inside-fMRI Non-switch, Switch and Switch cost behavioral performances. In summary, key behavioral outcome measures for task-switching ability in our study included the inside- and outside-fMRI behavioral performance for the Non-switch and Switch blocks, and for Switch cost, as well as the number of completed stages and total errors of the IED test. We considered the IED and outside-fMRI behavioral performances could better represent participant’s actual task-switching ability because the inside-fMRI task-switching performance may be influenced by ceiling effects, given that participants had practiced the non-switch and switch tasks and reached the ≥70% accuracy rate criterion before undertaking the fMRI scans.

### Behavioral and Physical Data Analyses

Between-group pre-intervention differences in demographics, physical tests, IED task-switching behavioral measure, and the inside- and outside-fMRI behavioral measures for the Non-switch, Switch, and Switch cost were analyzed by using the independent *t*-test. To analyze the effects of the TCC intervention on physical functions and social interaction, we performed separate two-way (group × time) RM ANOVAs on bilateral knee extensor strength, OLST, FSST, 6MWT and frequency of social interaction, with time as the within-subject factor. To understand the effects of the TCC intervention on task-switching behavioral outcomes, we performed separate two-way (group × time) RM ANCOVAs on the number of completed stages and total errors of the IED test, on the error rate of the outside- and inside-fMRI Non-switch and Switch conditions and Switch cost, and on the RT of the inside-fMRI Non-switch and Switch conditions and Switch cost, controlling for education, because education is known to influence task-switching performance (Zahodne et al., 2014). We adjusted *p*-value for multiple comparisons wherever needed. Because the correlations among the four physical measures (the knee extensor strength, OLST, FSST and 6MWT) ranged from −0.502 to 0.526, we adjusted the *p*-value to 0.0125 (= 0.05/4). The variable of frequency of social interaction had no significant correlation with these four physical variables (*r* ranged between −0.222 and 0.060; *p* > 0.05), therefore, this *p*-value was not adjusted and was set at *p* = 0.05. For the task-switching behavioral variables, because we used three behavioral variables (outside-fMRI error, inside-fMRI error and inside-fMRI RT) at the same time when we ran analyses of non-switch, switch, or switch cost performance and these variables were to some extent related (*r* ranged from 0.193 to 0.599 for baseline data), we adjusted the *p*-value to 0.017 (= 0.05/3). For the IED variables, because there was no significant relationship between the numbers of IED completed stages and IED total errors (*r* = −0.229, *p* = 0.216), suggesting no multicollinearity concern between these two variables, we did not adjust the *p*-value (*p*-value was set at 0.05) when examining the group and time effects on these two IED variables.

### Image Acquisition Parameters

All image data were acquired by using a 3-Tesla Trio MRI with a 32-channel head coil (Siemens Healthcare, Erlangen, Germany) at the National Taiwan University Hospital. Three types of brain images were collected for each participant: a T1-weighted image, using Magnetization-Prepared Rapid Acquisition Gradient Echo (repetition time (TR)/TE (echo time) 2,000 ms/2.98 ms, flip angle (FA) = 9°, field of view (FOV) = 192 × 256 mm^2^, coronal slice number = 208 slices, voxel size = 1 × 1 × 1 mm^3^); a T2-weighted image (TR/TE = 7,240 ms/101 ms, FA = 90°, FOV = 192 × 192 mm^2^, axial slice number = 34 slices, voxel size = 0.8 × 0.8 × 4 mm^3^); and two runs of T2* weighted echo planar image depicting BOLD contrast (TR/TE = 2,000 ms/24 ms, FA = 90°, axial slice number = 34 slices, FOV = 192 × 192 mm^2^, voxel size = 3 × 3 × 4 mm^3^).

### Image Preprocessing and Analysis

All preprocessing and general linear model (GLM) estimations were carried out using the Statistical Parametric Mapping 12 (SPM12; Wellcome Trust Centre for Neuroimaging, London, UK) implemented in MATLAB version 16.0 (The MathWorks, Natick, MA, USA). During image acquisition for each participant, each run of the fMRI scan started with a 6-s dummy scan in consideration of signal equilibrium and subject’ adaptation to imaging noise. These dummy scans were excluded from data analysis. For preprocessing of functional images, slice time correction and intra-session alignment were performed to spatially realign the images to the first image of each time series. Head motion correction was performed using a 6-parameter rigid body. Images with head motion greater than 3 mm in translation or 3° in rotation in any run were excluded, based on our scanning voxel resolution. Functional images were then co-registered to co-planar T2 images, which was then used for co-registration to T1-weighted images. Co-registered images were normalized to the Montreal Neurological Institute (MNI) template using the segmentation approach in SPM12, and then spatially smoothed using an 8-mm full-width at half-maximum Gaussian filter.

For first-level whole-brain analysis, each participant’s functional brain images for each session and each run were submitted to a GLM to estimate voxel-wise responses during physical size judgment, numerical magnitude judgment and switching across runs. Specifically, first-level GLMs included three regressors based on the vectors of onsets convolved with the hemodynamic responses function (HRF) for trials with correct responses in physical size blocks, numerical size blocks, and switch (both physical size and numerical magnitude together) blocks, and one regressor based on the onsets of all incorrect trials across all blocks convolved with the HRF. These four task regressors with six motion covariates and a constant for the mean run response were replicated over the two runs for each of the two sessions resulting in a total of 44 regressors in each participant’s first-level GLM. Individual contrast maps for Non-switch (average of numerical magnitude and physical size trial responses during physical size and numerical magnitude blocks relative to rest fixation) and Switch conditions (average of numerical magnitude and physical size trial responses during switch blocks relative to rest fixation) and Switch > Non-switch contrast were then generated and submitted as the dependent variable in a second-level random effects group analysis with group (TCC and CON) and time (pre- and post-intervention) as independent variables. The whole-brain analysis of the Switch > Non-switch contrast for each group and each time point was performed, and the threshold was set at a significance criterion of voxel-wise *p* < 0.005 corrected for FWE and a cluster size of at least 10 voxels. We then generated a disjunction activation map to identify voxels that showed significant Switch > Non-switch contrast in at least one of the groups for at least one of the time points (Smith et al., 2013). There are three reasons that we chose disjunction analysis. First, the disjunction method is fair to consider all brain functional activation sensitive to Switch > Non-switch contrast across groups and time points. Second, this method can reduce the multiple comparisons (within- and between-group contrasts) in fMRI data processing, and reduce the noise problem introduced from these multiple contrasts. By using the disjunction analysis and the RM ANCOVA with Bonferroni corrections, we could correct for multiple comparisons of the BOLD signals all at once. Third, we anticipated a relatively small difference in the changes of BOLD responses after the short-term TCC intervention and these small changes may not be discernable in group whole-brain contrast analysis that directly compares the significant differences of brain activation changes (post vs. pre) between the TCC and CON groups at the Switch > Non-switch contrast. Therefore, we chose the disjunction analysis and determined the critical ROIs for our analyses. Disjunctions were considered significance using the same threshold of voxel-wise *p* < 0.005 corrected for FWE and a cluster size of at least 10 voxels. Thus, a voxel was deemed to show a significant disjunction response if Switch > Non-switch contrast response passed the criterion for any group at any time point. We then evaluated BOLD response magnitude in functional regions-of-interest (ROIs) identified in this disjunction analysis.

### ROI Analysis

Because our *a priori* ROIs were in the PFC, we delineated functional ROIs as 5 mm radius spheres centered around voxels showing peak responses in prefrontal regions in the disjunction map generated above. For each participant, mean BOLD response magnitude for Non-switch and Switch conditions, and for Switch > Non-switch contrast, within each functional ROI were extracted using the Marsbar software^2^ (Brett et al., 2002). To evaluate the effect of TCC training on BOLD response magnitude in the *a priori* prefrontal ROIs, we first performed two-way ANCOVAs on BOLD response magnitudes in three identified ROIs for the Switch > Non-switch contrast with group and time as independent variables and age, gender, and years of education as the covariates. We adjusted the *p*-value to be 0.017 (=0.05/3) because there were three prefrontal ROIs. *Post hoc* tests with Bonferroni corrections were performed. We also evaluated the relationships between changes in BOLD response magnitude during the Switch condition and in task-switching behavioral measures across time. The changes were all calculated as post-intervention values minus pre-intervention values for corresponding variables. Specifically, we ran partial correlation analyses of changes in Switch BOLD response magnitude in the ROIs with changes in the number of total errors and completed stages of the IED test, changes in error rate of the outside-fMRI Switch condition, and changes in error rate and RT of the inside-fMRI Switch condition, and controlled for age, gender, and years of education, for the TCC and CON groups. We used the Switch BOLD response magnitudes for these correlation analyses in consideration of their comparability with the task-switching behavioral measures, which were taken from direct task-switching performance rather than Switch > Non-switch contrasts. All statistical analyses were performed in SPSS version 18.0.

## Results

### Participants

Two-hundred and eleven people who volunteered to participate in this study were initially screened. Among them, 31 participants were eligible and randomly assigned to the TCC (*N* = 16) or CON (*N* = 15) groups. No participants in the TCC group dropped out (i.e., 100% completion rate). Four participants in the CON group dropped out due to personal reasons (i.e., 73.3% completion rate). In addition, one CON participant had excessive head motion during the post-intervention fMRI scan. After exclusions due to incompletion (*N* = 4) and excessive head motion (*N* = 1), the final sample for data analyses was 26 (*N* = 16 for the TCC group and *N* = 10 for the CON group; Figure 1). Since this study was an RCT trial, we adopted the intention-to-treat analysis approach to treatment of data.

The baseline characteristics of the TCC and CON groups did not differ in age, education, body mass index, general cognitive function measured with MoCA, depression level measured with GDS-15, physical activity level measured with PASE, and frequency of social interaction activities (Table 1). There were no group differences in the results of pre-intervention physical function tests, number of completed stages and total errors of the IED test, the error rate of the outside-fMRI Non-switch and Switch conditions and Switch cost, and the error rate and RT of the inside-fMRI Non-switch and Switch conditions and Switch cost (all *p* values > 0.05).

**Table 1:** Demographics of the Tai Chi Chuan (TCC) and control (CON) groups at pre-intervention.

	TCC (*n* = 16)	CON (*n* = 15)	*p*
Age (years)	64.9 ± 2.8	64.9 ± 3.2	0.989
Age range (years)	61−69	60−69	
Gender (female:male)	13:3	15:0	
Education (years)	13.8 ± 2.4	13.4 ± 2.6	0.645
BMI (kg/m^2^)	22.5 ± 2.7	22.3 ± 3.2	0.863
MoCA (score)	28.3 ± 1.5	28.4 ± 1.5	0.868
GDS (score)	1.8 ± 2.0	1.9 ± 1.6	0.859
PASE (score)	50.3 ± 36.2	40.5 ± 18.4	0.352
Frequency of social interaction (times/week)	7.0 ± 2.0	7.1 ± 2.0	0.854

*Data are presented as means ± standard deviations. Independent t-test was used for all other group comparisons. BMI, body mass index; GDS, Geriatric Depression Scale; MoCA, Montreal Cognitive Assessment; PASE, Physical Activity Scale for the Elderly*.

In the last 2 weeks of the 12-week intervention, all participants in the TCC group learned the 24 forms of Yang-style TCC, by demonstrating the ability to execute the forms consecutively on their own as a group, in the absence of verbal instructions or cues from the coach. However, we did not rate individual skill level or fluency of movements because all these participants were novices to TCC before being enrolled into this study and thus could be considered as beginners of TCC only.

### Behavioral Performance

**Figure 3:**
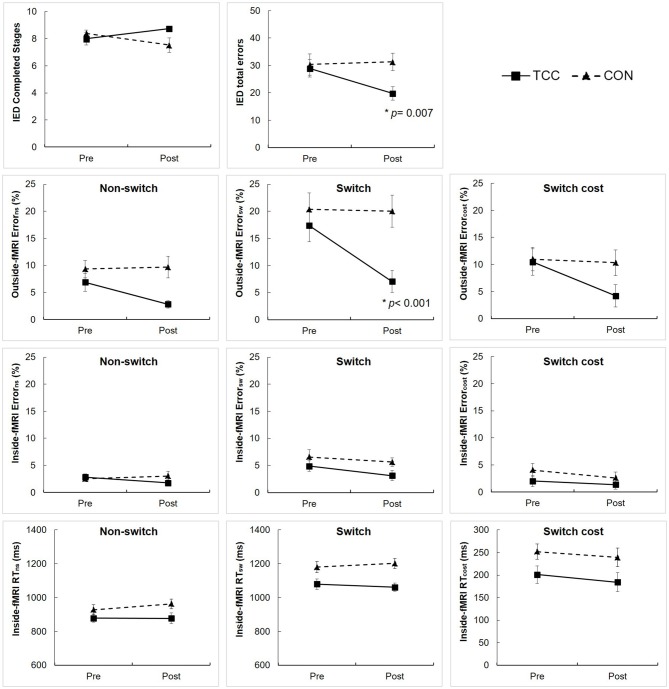
Intra-Extra Dimensional Set Shift (IED) performance and outside-fMRI and inside-fMRI Non-switch, Switch, and Switch cost performances of the Tai Chi Chuan (TCC) and control (CON) groups at the pre- and post-intervention tests. Values are means ± standard errors. There were no significant group differences at pre-intervention tests using independent *t*-test. We set *p* = 0.05 for the IED variables, and adjusted *p*-value = 0.017 for testing group × time interaction, group, and time effects on outside-fMRI and inside-fMRI Non-switch, Switch and Switch cost performances using RM ANCOVA. *Significantly different from pre-intervention in *post hoc* analysis. cost, value in Switch condition − value in Non-switch condition; Error, error rate; ns, Non-switch condition; sw, Switch condition.

Results of the two-way RM ANCOVA analysis showed significant group × time interaction effects on the number of total errors of the IED performance (*F*_(1,28)_ = 4.93, *p* = 0.035) and error rate of the outside-fMRI Switch condition (*F*_(1,28)_ = 16.86, *p* < 0.001; Figure 3). *Post hoc* tests revealed that the TCC group, but not the CON group, significantly reduced the number of total errors of the IED performance (*t*_(15)_ = 3.13, *p* = 0.007) and the error rate of the outside-fMRI Switch condition (*t*_(15)_ = 6.34, *p* < 0.001) from pre- to post-intervention (Figure 3). There was a marginal interaction effect on the number of completed stages of the IED performance (*F*_(1,28)_ = 4.16, *p* = 0.051), error rate of the outside-fMRI Non-switch condition (*F*_(1,28)_ = 3.99, *p* = 0.056) and Switch cost contrast (*F*_(1,28)_ = 3.31, *p* = 0.079) with the TCC group showing a trend of increasing the number of completed stages of the IED test and reducing the error rate of the outside-fMRI Non-switch condition and Switch cost contrast, whereas the CON group lacking changes (Figure 3). There were no significant interaction effects on other behavioral measures of the inside-fMRI Non-switch, Switch, and Switch cost (Supplementary Table S1).

**Figure 4:**
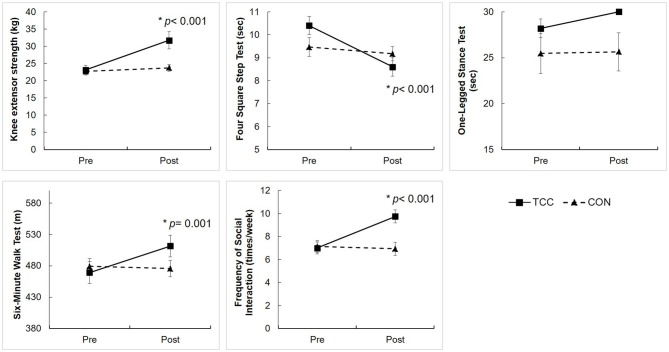
Physical function performance and frequency of social interaction of the TCC and CON groups at the pre- and post-intervention tests. Values are means ± standard errors. We set adjusted *p*-value = 0.0125 for testing group × time interaction, group, and time effects on four physical variables (knee extensor strength, Four Square Step Test (FSST), One-legged stance test (OLST), 6-Minute Walk Test (6MWT), and set *p*-value = 0.05 for testing group × time interaction, group, and time effects on frequency of social interaction using RM ANCOVA. *Significantly different from pre-intervention in *post hoc* analysis. There were no significant group differences at pre-intervention tests, using independent *t*-test.

The two-way RM ANOVA analysis on physical function and social interaction showed significant group × time interaction effects on knee extensor strength (*F*_(1,29)_ = 13.52, *p* = 0.001), the FSST (*F*_(1,29)_ = 14.52, *p =* 0.001), the 6MWT (*F*_(1,29)_ = 12.70, *p* = 0.001), and the frequency of social interaction (*F*_(1,29)_ = 26.62, *p* < 0.001). The *post hoc* tests revealed that the TCC group improved on knee extensor strength (*t*_(15)_ = −4.54, *p* < 0.001), FSST (*t*_(15)_ = 5.88, *p* < 0.001), 6MWT (*t*_(15)_ = −3.98, *p* = 0.001), and frequency of social interaction (*t*_(15)_ = −5.37, *p* < 0.001), but the CON group did not (Figure 4). There was no significant interaction on OLST (*F*_(1,29)_ = 2.25, *p* = 0.145).

### Task-Switching fMRI Activation

**Figure 5:**
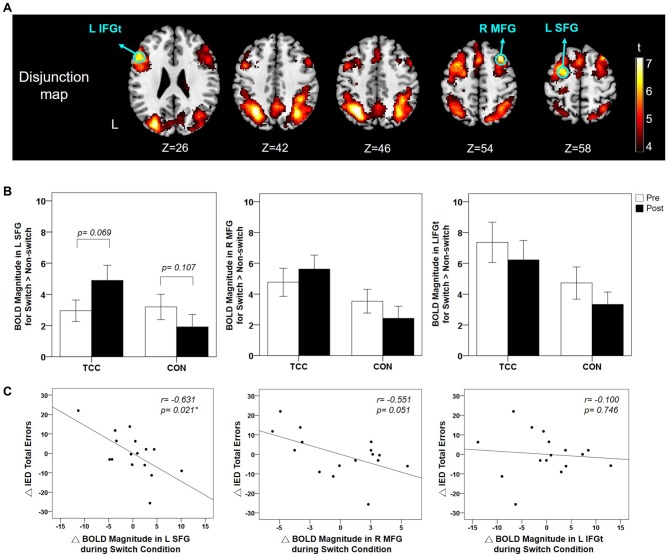
**(A)** Disjunction map of the Switch > Non-switch contrast across groups and time points (voxel-wise *p* < 0.005 with FWE correction). The locations of the functional ROIs in the prefrontal regions are indicated using green colored circles. **(B)** Mean and standard errors of BOLD response magnitude in these three functional ROIs for Switch > Non-switch contrast for the TCC and CON groups at the pre- and post-intervention scans. For the left SFG, the group × time interaction effect was significant (*p* = 0.017) with the TCC group showing a marginal increase (*p* = 0.069) in BOLD response magnitude in Switch > Non-Switch contrast after training in contrast to a non-significant change in the CON group. **(C)** Partial correlation plots showing that the changes of BOLD response magnitude during the Switch condition in the left SFG and right MFG ROIs significantly (*p* = 0.021) and marginally (*p* = 0.051) correlated with the changes of the number of total errors of the IED test from pre- to post-intervention tests for the TCC group, respectively, after controlling for age, gender, and education. ΔBOLD, post-intervention BOLD value − pre-intervention BOLD value; ΔIED total errors, post-intervention number of IED total errors − pre-intervention number of IED errors; BOLD, blood oxygenation level dependent; IED, Intra-Extra Dimensional Set Shift; L SFG, left superior frontal gyrus; R MFG, right middle frontal gyrus; L IFG_t_, left inferior frontal gyrus pars triangularis. *Significant correlation between ΔIED total errors and ΔBOLD, *p* < 0.05.

The functional activations of the Switch > Non-switch contrast per group and per time point (Supplementary Figure S1) were submitted to a disjunction analysis (see “Materials and Methods” section). The resulting disjunction map identified functional ROIs over the left SFG, IFG_t_, inferior parietal gyrus, and right MFG and angular gyrus (Table 2). These regions showed significant activation for Switch > Non-switch contrast in at least one of the groups for at least one of the time points. We further analyzed the BOLD response magnitudes in the *a priori* prefrontal functional ROI regions- the left SFG, left IFG_t_, and right MFG (Figure 5A). We adjusted *p*-value to be 0.017 (= 0.05/3) for multiple comparisons of the BOLD response across group and time, and controlled for age, gender, and education. Two-way RM ANCOVA analysis on the mean BOLD response magnitude of Switch > Non-switch response contrast in these three ROIs revealed a significant group × time interaction effect in the left SFG (*F*_(1,26)_ = 6.57, *p* = 0.017) and a marginal significant interaction effect in the right MFG (*F*_(1, 26)_ = 3.31, *p* = 0.081), but no interaction effects in the left IFG_t_ (Supplementary Table S2). *Post hoc* tests qualified that the left SFG interaction was due to marginally increased Switch > Non-switch BOLD response contrast in the TCC group (*t*_(15)_ = −1.96, *p* = 0.069, two-tailed) but a non-significant difference in the CON group (*t*_(14)_ = 1.72, *p* = 0.107, two-tailed) from the pre- to post-intervention scans (Figure 5B). Overall, the CON group showed a non-significant trend of decreased Switch > Non-switch neural response contrast in all three ROIs from the pre- to post-intervention scans (*p* > 0.05). In sum, after a 12-week intervention, the TCC group showed a trend of increased Switch > Non-switch BOLD response contrast in the left SFG and right MFG ROIs, whereas the CON group showed a trend of decreased Switch > Non-switch BOLD response contrast in all three ROIs. There were no regions showing significant functional activation for the reverse Non-switch > Switch contrast.

**Table 2:** Peak Montreal Neurological Institute (MNI) coordinates and activation details in frontoparietal regions identified in the disjunction map of the Switch > Non-switch contrast across groups and time points.

Brain area	*x*	*y*	*z*	*T*	No. of voxels
L Superior Frontal Gyrus	−22	−4	58	6.48	238
R Middle Frontal Gyrus	32	18	54	7.39	54
L Inferior Frontal Gyrus pars Triangularis	−50	20	26	6.72	245
L Inferior Parietal Gyrus	−32	−60	46	7.92	744
R Angular Gyrus	32	−64	42	6.95	283

*Whole-brain analysis with threshold set at voxel-wise *p* < 0.005 corrected for FWE and a cluster size at least 10 voxels. Positive t-values indicate stronger activation in the Switch condition than in the Non-switch condition*.

### Associations Between Task-Switching fMRI Brain Activation and Behavioral Performance

Results of partial correlation analysis between task-switching fMRI brain activation and behavioral performance for the TCC group showed negative moderate-to-good correlations between the changes in Switch BOLD response magnitude in the left SFG (*r* = −0.631, *p* = 0.021) and a marginal significant correlation in the right MFG (*r* = −0.551, *p* = 0.051) during the fMRI experiment with changes in the number of total errors of IED test (Figure 5C), suggesting that TCC participants who had greater activation increments in the left SFG and right MFG regions during the Switch condition also showed greater reductions of the number of total errors on the IED test (i.e., improved task-switching performance) after training. There were no other significant correlations between task-switching fMRI brain activation and other task-switching behavioral measures (all *p* values > 0.05; Supplementary Table S3) for the TCC group. As to the CON group, there was no significant correlation between any changes in task-switching fMRI brain activation and any changes in task-switching behavioral measures (all *p* values > 0.05; Supplementary Table S4).

## Discussion

To our knowledge, our study is the first RCT to investigate TCC training-induced changes in brain functional activation during a task-switching fMRI experiment in cognitively intact older adults (Yu et al., 2018) and the correlations between these functional neural processing changes and changes in task-switching performance. Behaviorally, the 12-week TCC training led to improved cognitive task-switching ability, as indicated by the reduced number of total errors on the IED test and the reduced error rate of the outside-fMRI Switch performance. The TCC training also resulted in improved physical performance, including muscle strength, mobility, and cardiorespiratory endurance and better social interaction. With regard to changes of brain functional activation, we found that after the intervention period, the TCC group presented a marginally increased functional engagement and a trend of increased engagement in the left SFG and the right MFG (bilateral DLPFC), respectively, for the Switch > Non-switch contrast; whereas the CON group showed a trend of decreased engagement of all three prefrontal ROI regions. More importantly, individuals who showed greater increments of such DLPFC engagement had greater improvement in task-switching performance assessed with the IED test. These novel findings regarding functional brain activation provided evidence in support of enhanced DLPFC functional processing as a possible mechanism for the role of TCC training in modulating older adult task-switching performance.

### Effects of TCC Training on Behavioral Performance

Among the three task-switching behavioral measures, we found that after TCC training, the TCC group showed a reduced number of total errors on the IED test, and a reduced error rate of the outside-fMRI Switch performance, but not during the actual fMRI scans. We considered participants’ performance on the outside-fMRI practice session as important outcome measures of their actual task-switching abilities because there were no practice influences on this performance. In contrast, participants underwent the fMRI scans after they had practiced 40 Non-switch and 40 Switch trials and reached *a priori* criterion of accuracy (≥70%), which could introduce practice-induced ceiling effects on the inside-fMRI task-switching performance and make this performance less representative of participant’s actual task-switching ability. Indeed, when we ran paired-*t* tests on the Non-switch and Switch performances between the outside-fMRI and the inside-fMRI trials for the pre-intervention data, we found significantly smaller number of errors for both Non-switch and Switch performances on the inside-fMRI trials (*p =* 0.039 and 0.001, respectively, for the TCC group; and both *p* < 0.001 for the CON group). These significant differences suggested practice effects on the inside-fMRI performance. Furthermore, the IED test, designed to specifically tax the cognitive processes of shifting attention between different dimensions of visual stimuli, is a reliable and valid clinical instrument for measuring aspects of cognitive flexibility among executive function (Robbins et al., 1998). It has been shown that the number of total errors on the IED test has a moderate correlation with that on the Wisconsin Card Sorting Test (Kim et al., 2014), one of the gold standard neuropsychological tests for “set-shifting” or “task-switching” (Monchi et al., 2001). Therefore, our findings that the TCC group showed improvements on the IED test and the outside-fMRI Switch performance after training support the notion that TCC training has beneficial effects on task-switching behavioral performance in older adults.

In past research based on the TMT-B, it was difficult to tease out whether the beneficial effects of TCC training were on task-switching, working memory, or both (Matthews and Williams, 2008; Mortimer et al., 2012; Nguyen and Kruse, 2012). Our findings provided more direct evidence in support of the effects of TCC training on task-switching vis-à-vis our examination of effects on switch cost (Switch > Non-switch) rather than just non-switch or switch neural responses. While speculative, these beneficial effects on task-switching may stem from the requirement during TCC practice that participants smoothly shifted from one form of TCC to the next till the end of a total of 24 forms. Such drills may not only enhance their sustained attention and memory, but also the task-switching abilities (Chang et al., 2014; Fong et al., 2014; Zheng et al., 2015). Future experiments that break down the components of TCC training to evaluate each of their effects are needed to precisely isolate the specific mechanisms of TCC training in changing cognitive and neural processing.

Non-cognitive aspects of our findings consistent with past studies on TCC training should also be highlighted. Regarding physical performance, our findings that the TCC group improved their lower extremity extensor strength, mobility, and cardiorespiratory endurance are congruent with previous research (Li et al., 2001; Taylor-Piliae et al., 2006; Taylor-Piliae, 2008; Lan et al., 2013). Such improvements in physical performances might be attributed to the requirement to maintain a semi-squat posture while performing whole body movements in a continual manner during TCC practice (Lan et al., 2013). However, different from previous literature, we did not find TCC training effects on static balance performance- the OLST. Note that the majority of our participants already had good balance ability before training, with 81% of the TCC and 73% of the CON participants showing a score of 30 s on the OLST before training. More difficult tests may be needed to sensitively detect the effects of TCC training on balance ability in these participants. Finally, our results also showed that the TCC group increased the frequency of social interactions after training, supporting previous literature which revealed that practicing TCC enhances social support (Yeh et al., 2016; Chan et al., 2017). Together, these positive TCC training effects on cognitive and physical functions, and social interactions indicate that regular engagement in a TCC program three times a week for 3 months is sufficient to induce detectable cognitive and physical health benefits and enhance social support in cognitively intact older individuals.

### Effects of TCC Training on Task-Switching Related Brain Activation

Our disjunction analysis of fMRI activations in the *a priori* prefrontal regions showed that the left SFG, right MFG, and left IFG_t_ were the main regions activated for Switch > Non-switch contrast among the participants. These identified prefrontal regions implicated in our task-switching number Stroop experiment were consistent with those reported in older adults in prior task-switching studies (DiGirolamo et al., 2001; Zhu et al., 2014; Hakun et al., 2015). Our results provided two lines of evidence supporting that the two task-switching specific DLPFC regions were particularly modulated after TCC training. First, after TCC training, the TCC group presented a marginally increased functional activation in the left SFG and a trend of increased activation in the right MFG for Switch > Non-switch contrast. Second, after TCC training, those participants who showed greater increases of activation in these two regions during task-switching presented better improvement on the IED test performance. These findings suggested that the left SFG and right MFG activation was specifically modulated during task-switching after TCC training to enhance task-switching performance in some, although not all, of the TCC participants. Intriguingly, previous research that used resting-state fMRI to investigate effects of a 6-week multimodality intervention program, comprising TCC, cognitive training and group counseling, on resting-state brain activation also revealed modulation of brain activation in these two DLPFC regions (Yin et al., 2014). Yin et al. (2014) found significant increases in the resting-state brain activation in the left SFG and right MFG for participants who received the multimodality intervention program and that greater increases of the activation in the right MFG were correlated with greater improvements in TMT performance. Furthermore, using structural brain imaging, Wei et al. (2013) found that TCC experts with an average of 14-year TCC experiences had thicker cortex in right MFG as compared to age-matched TCC-naive control individuals. Together with our findings, we speculate that in some older adults, TCC training may specifically enhance neural processing in the left SFG and right MFG regions during resting and during task-switching and enlarge these regions, which in turn, could lead to more efficient task-switching performance. The three key cognitive processes of task-switching entail control of attention maintenance, inhibition and working memory (Monsell, 2003). The left SFG is known to be involved in the implementation of top-down executive control by maintaining attention demands while switching between tasks (MacDonald et al., 2000) and contributing to working memory processing, such as loading, monitoring, and maintaining different stimulus-response rules (du Boisgueheneuc et al., 2006; Cutini et al., 2008). The right MFG is associated with inhibition control of ignoring irrelevant stimuli or inhibiting undesirable responses (Garavan et al., 1999; Brass et al., 2001; Buchsbaum et al., 2005). The fact that while practicing TCC, participants have to memorize a series of whole-body movements, maintain attention on coordinating breath and actions, and switch from one Tai Chi form to another smoothly may provide extensive drills on attention, working memory, and inhibition control and thereby enhances the engagement of the left SFG and right MFG regions and makes the activation in these regions more efficient.

It is worth noting that our study showed that except for the left SFG region, TCC practice did not significantly increase brain activations in other prefrontal regions engaged in task-switching across all TCC practitioners. However, among the TCC practitioners, those who became better at increasing the prefrontal activations during task-switching could significantly reduce task-switching behavioral errors after TCC training. These findings suggested individual differences in their brain responses to short-term practice of TCC, which may potentially provide benefits to some, although not all, older adults to enhance the function of their prefrontal activations during task-switching. It remains to be studied whether a longer-term of TCC training could bring more homogenous positive effects on prefrontal activation to all TCC practitioners. Another possible account of the small amount of prefrontal activation changes after TCC training could be the practice-induced ceiling effects resulting from participants’ practice of 40 non-switch and 40 switch trials outside the MRI scanner in order to ensure their fully understanding of the tasks and reaching the 70% performance accuracy criterion before being scanned. As such, the prefrontal activation changes due to TCC training *per se* may be attenuated and hence become less discernible. Nevertheless, we suggest that even if ceiling performance is reached behaviorally for the inside-MRI task because of the practice performance criterion, neural responses can still reflect level of ease or difficulty to reach the ceiling response, as seen with the correlations with the out of scanner IED performance.

We note that our finding of a non-significant trend of decreasing activation during task-switching activation in prefrontal ROIs for the CON group is consistent with previous studies which showed a non-significant trend for brain volume reduction of the control group who did not receive any exercise training (Mortimer et al., 2012). These findings suggest that normative age-related changes in brain structure and function might be observed in as little as 3 months, albeit not significant in our study. More importantly, we provide evidence suggesting that TCC training not only halts but reverses this normative trajectory of brain functional aging in target frontal brain areas in some older adults.

While we speculate that TCC practice particularly challenges learners’ task-switching processing, and thereby improves learners’ task-switching abilities, it remains possible that the TCC training effects on task-switching may come from the aerobic or social interaction effects of TCC practice (Li et al., 2001; Taylor-Piliae, 2008; Mortimer et al., 2012; Lan et al., 2013; Yeh et al., 2016; Chan et al., 2017; Ma et al., 2018). Aerobic training studies showed that enhanced cardiorespiratory fitness after such training was associated with improvements in cognitive function (Colcombe and Kramer, 2003; Smith et al., 2010) and changes in brain activation (Colcombe et al., 2004; Voelcker-Rehage et al., 2011). Participation in social activities is known to be associated with better cognitive function or protection against cognitive decline in older adults (Glei et al., 2005; Mortimer et al., 2012; Hikichi et al., 2017; Fu et al., 2018). Since we found our TCC participants had improvements on the knee extensor strength, mobility (FSST), cardiorespiratory fitness (6MWT) performance and social interaction, we further performed correlation analyses of TCC participants’ changes on these physical and social interaction measures with changes in activation in the three identified prefrontal ROIs during task-switching and changes in task-switching behavioral measures, using age, gender, and education as the covariates. The analyses yielded no significant relationships of task-switching abilities with physical measures (all *p* > 0.05; Supplementary Table S3), suggesting that it is unlikely that the improvements of task-switching behavioral performance after TCC training came from the physical effects of TCC. However, we found a significant correlation between the change in social interaction and the change in the outside-fMRI error rate of switch condition (*r* = −0.690; *p* = 0.009), and between the change in the knee extensor strength and the change in the Switch BOLD response magnitude of the left IFG_t_ (*r* = 0.802; *p* = 0.001) for the TCC group (Supplementary Table S3). To further examine whether changes in social interaction influenced the relationships between changes in the outside-fMRI error rate of the switch condition and changes in the Switch BOLD response magnitude for the three ROIs, we re-ran the partial correlation analyses using age, gender, education, and social interaction as the covariates. The results remained the same and there were still no significant correlations between changes in the outside-fMRI error rate of the switch condition with changes in Switch BOLD response for these three ROIs (*r* = −0.324–0.148, *p* > 0.05; Supplementary Figure S2). Similarly, to further test whether the change in the knee extensor strength affected the relationships between changes in task-switching performance and Switch BOLD response magnitude in the left IFG_t_, we controlled for age, gender, education, and the knee extensor strength as covariates. The results also showed no significant relationships between changes of all task-switching measures with changes in the Switch BOLD response magnitude in the left IFG_t_ (*r* = −0.081–0.403, *p* > 0.05; Supplementary Figure S3). According to these results, it is unlikely that the newly emerged relationships between changes in brain activation and changes in switch performance after TCC training came from the improved social interaction or knee extensor strength for the TCC group. Rather, it is more possible that practicing TCC provides specific drills on cognitive processes needed for task-switching, and therefore leads to improved task-switching performance in most practitioners and altered neural activation in brain regions particularly engaged in task-switching in some practitioners. However, because we only performed the 6MWT and did not measure the maximum cardiorespiratory capacity, such as maximal VO_2_ uptake, as reported in previous studies using aerobic exercises (Colcombe et al., 2004; Voelcker-Rehage et al., 2011), it remains to be tested as to whether TCC participants’ changes in the maximum cardiorespiratory capacity relate to their changes in brain activations after TCC practice.

### Comparisons of Brain Mechanisms of Cognitive Effects Between TCC and Other Types of Exercises

We note that there are similarities and differences in the effects of TCC training on cognition and neural processing, compared to other forms of exercises including aerobic, resistance and cognitive-motor exercises. Aerobic or resistance exercises have been shown to improve inhibition control (Colcombe et al., 2004; Voelcker-Rehage et al., 2011; Liu-Ambrose et al., 2012; Nagamatsu et al., 2012), but not task-switching components of executive function (Liu-Ambrose et al., 2010; Voss et al., 2013) in cognitively intact older adults. By contrast, cognitive-motor exercises, a form of dual-task training comprising simultaneous cognitive and motor loads (Wollesen and Voelcker-Rehage, 2013), show positive effects on task-switching performance (Nishiguchi et al., 2015; Eggenberger et al., 2016), as well as greater spatial ability and working memory capacity compared to aerobic exercise (Moreau et al., 2015). These findings suggest that complex cognitive-motor training protocols are more effective in enhancing cognitive functions than simple motor training alone, consistent with the effects of TCC training in our study as well as others (Mortimer et al., 2012; Wayne et al., 2014).

Less consistency is seen in the effects of exercise interventions on brain responses during cognitive processing. Whereas some report aerobic or resistance exercise intervention increased prefrontal activation during a Flanker or associative memory task (Colcombe et al., 2004; Voelcker-Rehage et al., 2011; Liu-Ambrose et al., 2012; Nagamatsu et al., 2012), others report decreased prefrontal activation during similar tasks (Voelcker-Rehage et al., 2011; Smith et al., 2013). Decreased neural cognitive processing after training might reflect improved neural processing efficiency, whereas increased neural activity might reflect more specialized and enhanced cognitive operations. Thus, both types of exercise training related changes in neural processing can theoretically support improved cognition. However, we note that in our study, increases in prefrontal activity correlated with better task-switching ability in the TCC group, suggesting the latter enhancement of neural computations as the more likely outcome of TCC training effects on task-switching processing. Since TCC training also comprises the aerobic and resistance training components, it remains to be studied whether TCC training also improves inhibition and memory functions, and the associated neural mechanisms involved. Future studies directly comparing TCC training effects against aerobic/resistance exercises across different cognitive abilities and associated brain responses are required to evaluate the specific neural mechanisms that underlie the cognitive benefits afforded by TCC training.

### Limitations

Four limitations are noted in this present study. First, there was a relatively small sample size and a higher dropout rate for the CON than TCC group (26.7% in CON group v.s. 0% in TCC group; chi square, *p* = 0.022). The latter was possibly due to the lack of strong incentive to adhere to the study enrollment for the CON participants. Nevertheless, the independent *t*-test on baseline demographics and behavioral performance between the initial 15 CON participants and the remaining 10 CON participants yielded no significant group differences (all *p* > 0.05). Therefore, it is unlikely that the higher dropout rate in the CON group affected our results. Second, there were fewer male participants than female participants in this study, which could decrease the generalizability of the results to the general older population. Third, there was a lack of an active control group that performed another type of exercise during the intervention period. Adding an active control group could help differentiate whether our findings were due to TCC training *per se* from being engaged in any form of physical exercise. Fourth, this study only assessed the training effect immediately following the 12-week TCC training. Future research may extend the current findings by adding follow-up assessments after the end of training to explore the longer time influences and retention effects of a 12-week TCC program.

## Conclusion

This study demonstrated that 12 weeks of TCC training improved task-switching ability and induced a positive relationship between changes in task-switching associated prefrontal activation and improvement in task-switching performance in older adults. These findings suggest TCC training could potentially provide benefits to some, although not all, older adults to enhance the function of their prefrontal activations during task-switching. Future studies involving a longer-term of training and using examinations of brain structural and functional connectivity are possible next steps to further understand whether a longer-term of TCC training could bring more homogenous positive effects on brain prefrontal activation to all TCC practitioners and the mechanisms underlying improved neural processing after TCC intervention in those who benefit from the training.

## Author Contributions

M-TW and P-FT contributed to the conception of the work. M-TW, P-FT, JG, M-JC, Y-KC, T-LC, SS-FG and CL contributed to the design of the work. M-TW, Y-CH, Y-JC and N-CC contributed to the data acquisition and analysis. M-TW, P-FT, JG, Y-KC, T-LC and CL contributed to interpretation of data. M-TW and P-FT completed the first draft of the manuscript. All authors were involved in the manuscript revision and agreed with final approval of the version, and ensured the accuracy of investigation.

## Conflict of Interest Statement

The authors declare that the research was conducted in the absence of any commercial or financial relationships that could be construed as a potential conflict of interest. The reviewer C-HW and handling Editor declared their shared affiliation at the time of the review.

## Footnotes

^1^https://clinicaltrials.gov/ct2/show/NCT02270320

^2^http://marsbar.sourceforge.net
