# Supplementary figures and images for: Task-Switching Performance Improvements After Tai Chi Chuan Training Are Associated With Greater Prefrontal Activation in Older Adults
Source: Front Aging Neurosci. 2018 Sep 24;10:280. doi: 10.3389/fnagi.2018.00280 (PMC6165861; doi:10.3389/fnagi.2018.00280)

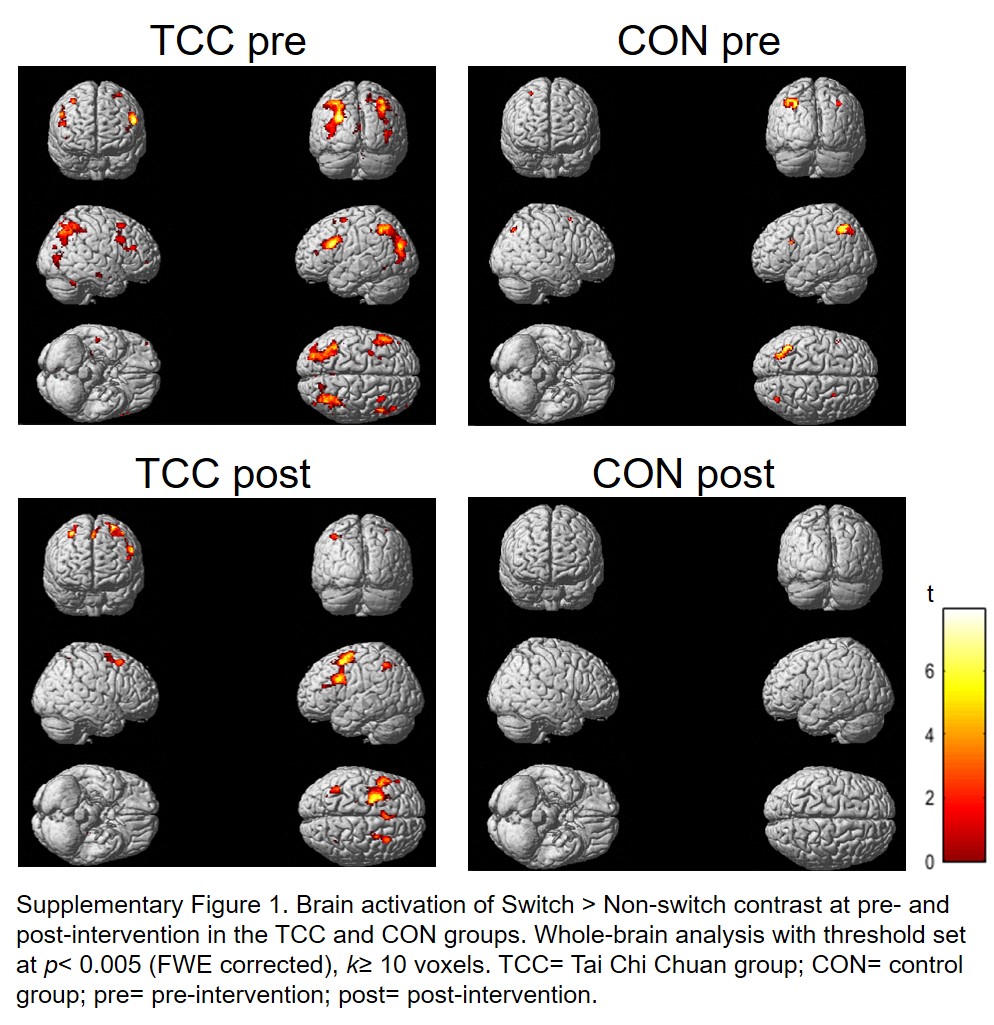

Supplement: Supplementary file 1 [file Image_1.JPEG]

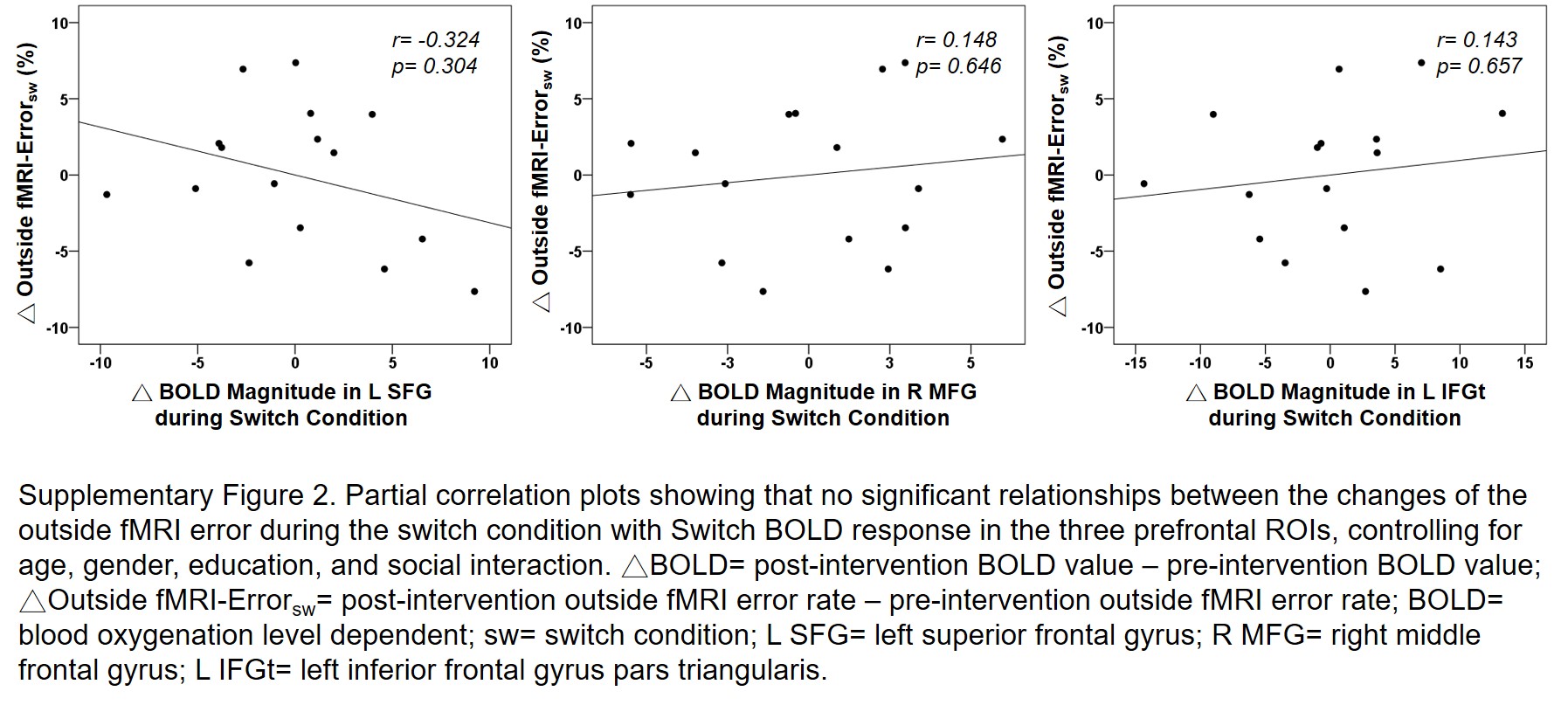

Supplement: Supplementary file 2 [file Image_2.JPEG]

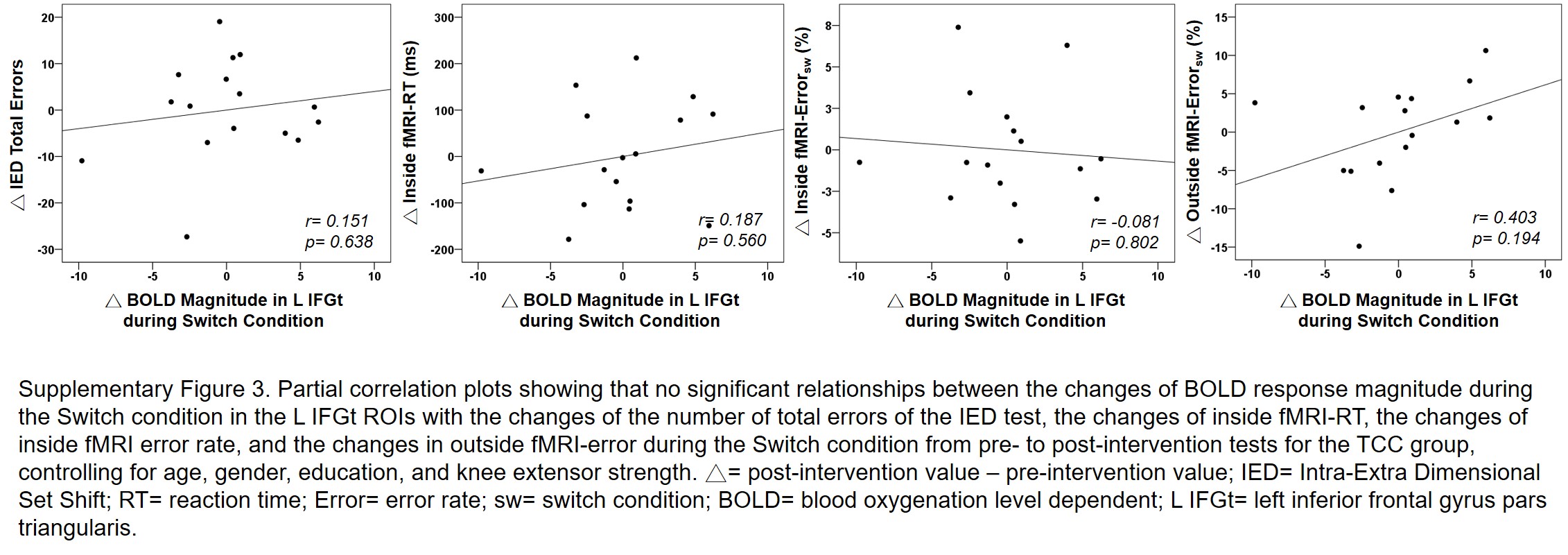

Supplement: Supplementary file 3 [file Image_3.JPEG]
