# Supplementary material for: Task-Switching Performance Improvements After Tai Chi Chuan Training Are Associated With Greater Prefrontal Activation in Older Adults
Source: Front Aging Neurosci. 2018 Sep 24;10:280. doi: 10.3389/fnagi.2018.00280 (PMC6165861; doi:10.3389/fnagi.2018.00280)
Supplement: Supplementary file 4 [file Table_1.DOCX]

**Supplementary Table 1. Outside- and inside- fMRI Non-switch, Switch, and Switch cost performance (error and RT) of the TCC and CON groups at pre- and post-intervention tests.**

|  | TCC (N= 16) | |  | CON (N= 15) | | Group × Time | Group | Time |
| --- | --- | --- | --- | --- | --- | --- | --- | --- |
|  | Pre-intervention | Post-intervention |  | Pre-intervention | Post-intervention |  |  |  |
| **Non-switch** |  |  |  |  |  |  |  |  |
| Outside-fMRI error (%) | 6.9 ± 6.6 | 2.8 ± 2.7 |  | 9.3 ± 6.2 | 9.7 ± 7.7 | 0.056 | 0.021 | 0.168 |
| Inside-fMRI error (%) | 2.8 ± 2.4 | 1.8 ± 2.1 |  | 2.3 ± 1.5 | 3.1 ± 3.9 | 0.096 | 0.728 | 0.363 |
| Inside-fMRI RT (ms) | 878.5 ± 96.4 | 877.5 ± 125.1 |  | 897.4 ± 95.7 | 949.8 ± 98.6 | 0.176 | 0.282 | 0.962 |
| **Switch** |  |  |  |  |  |  |  |  |
| Outside-fMRI error (%) | 17.3 ± 11.7 | 7.0 ± 8.1^†^ |  | 20.3 ± 11.9 | 20.0 ± 11.4 | < 0.001* | 0.046 | 0.642 |
| Inside-fMRI error (%) | 4.9 ± 3.9 | 3.1 ± 3.8 |  | 6.6 ± 6.0 | 5.2 ± 3.0 | 0.849 | 0.237 | 0.711 |
| Inside-fMRI RT (ms) | 1079.4 ± 119.3 | 1061.5 ± 93.9 |  | 1147.9 ± 122.8 | 1181.4 ± 112.2 | 0.224 | 0.042 | 0.687 |
| **Switch cost** |  |  |  |  |  |  |  |  |
| Outside-fMRI error (%) | 10.5 ± 9.8 | 4.2 ± 8.3 |  | 11.0 ± 8.3 | 10.3 ± 9.2 | 0.079 | 0.263 | 0.467 |
| Inside-fMRI error (%) | 2.1 ± 4.0 | 1.4 ± 3.4 |  | 4.2 ± 5.4 | 2.0 ± 4.5 | 0.515 | 0.255 | 0.889 |
| Inside-fMRI RT (ms) | 201.0 ± 76.5 | 184.0 ± 84.3 |  | 250.5 ± 62.4 | 231.6 ± 82.0 | 0.904 | 0.086 | 0.722 |

Values are means ± standard deviations. Outside-fMRI error means performance during practice trials. error= error rate; RT= reaction time.

^*^ adjusted *p*< 0.017: showing a significant difference, using RM ANCOVA and controlling for education. ^†^*p*< 0.001: post hoc analysis of repeated measures ANCOVA, showing a significant difference from pre-intervention test data.
