# Supplementary material for: Task-Switching Performance Improvements After Tai Chi Chuan Training Are Associated With Greater Prefrontal Activation in Older Adults
Source: Front Aging Neurosci. 2018 Sep 24;10:280. doi: 10.3389/fnagi.2018.00280 (PMC6165861; doi:10.3389/fnagi.2018.00280)
Supplement: Supplementary file 5 [file Table_2.DOCX]

**Supplementary Table 2. BOLD response magnitude for the Switch > Non-switch contrast of the TCC and CON groups at pre- and post- intervention**

|  | TCC (N= 16) | |  | CON (N= 15) | | Group × Time | Group | Time |
| --- | --- | --- | --- | --- | --- | --- | --- | --- |
|  | Pre-intervention | Post-intervention |  | Pre-intervention | Post-intervention |  |  |  |
| L SFG | 3.0 ± 2.8 | 4.9 ± 3.9 |  | 3.2 ± 3.2 | 1.9 ± 3.1 | 0.017* | 0.157 | 0.563 |
| R MFG | 4.8 ± 3.6 | 5.6 ± 3.6 |  | 3.5 ± 3.0 | 2.4 ± 3.0 | 0.081 | 0.048 | 0.876 |
| L IFGt | 7.4 ± 5.2 | 6.2 ± 5.0 |  | 4.7 ± 4.0 | 3.3 ± 3.1 | 0.764 | 0.096 | 0.742 |

Values are means ± standard deviations. ^*^adjusted *p*≤ 0.017: showing a significant difference using RM ANCOVA, controlling for age, education, and gender. L SFG= left superior frontal gyrus; R MFG= right middle frontal gyrus; L IFGt= left inferior frontal gyrus pars triangularis.
