# Supplementary material for: Task-Switching Performance Improvements After Tai Chi Chuan Training Are Associated With Greater Prefrontal Activation in Older Adults
Source: Front Aging Neurosci. 2018 Sep 24;10:280. doi: 10.3389/fnagi.2018.00280 (PMC6165861; doi:10.3389/fnagi.2018.00280)
Supplement: Supplementary file 6 [file Table_3.docx]

**Supplementary Table S3. Inter-relationships among changes in task-switching performance, changes in physical function and social interaction, and changes in BOLD response magnitude in the prefrontal cortex during the Switch condition from pre-intervention to post-intervention in the TCC group**

|  | △ IED Completed Stages | △ IED  Total Errors | △ Outside- fMRI Error_sw_ (%) | △ Inside- fMRI Error_sw_ (%) | △ Inside- fMRI RT_sw_ (ms) | △ Frequency of social interaction | △ BOLD  in L SFG | △ BOLD  in R MFG | △ BOLD  in L IFG_t_ |
| --- | --- | --- | --- | --- | --- | --- | --- | --- | --- |
| △ Knee extensor strength (kg) | *r*= 0.041  *p*= 0.895 | *r*= -0.234  *p*= 0.442 | *r*= -0.123  *p*= 0.689 | *r*= 0.134  *p*= 0.663 | *r*= -0.086  *p*= 0.781 | *r*= 0.110  *p*= 0.721 | *r*= 0.458  *p*= 0.116 | *r*= 0.314  *p*= 0.296 | *r*= 0.802  *p*= 0.001* |
|  |  |  |  |  |  |  |  |  |  |
| △ Four Square Step Test (sec) | *r*= 0.199  *p*= 0.514 | *r*= 0.075  *p*= 0.808 | *r*= 0.128  *p*= 0.676 | *r*= -0.093  *p*= 0.762 | *r*= -0.053  *p*= 0.863 | *r*= -0.237  *p*= 0.435 | *r*= -0.307  *p*= 0.308 | *r*= 0.048  *p*= 0.876 | *r*= -0.210  *p*= 0.492 |
|  |  |  |  |  |  |  |  |  |  |
| △ Six Minute Walk Test (m) | *r*= -0.087  *p*= 0.778 | *r*= 0.066  *p*= 0.830 | *r*= 0.316  *p*= 0.292 | *r*= -0.346  *p*= 0.247 | *r*= -0.247  *p*= 0.415 | *r*= -0.216  *p*= 0.479 | *r*= 0.213  *p*= 0.485 | *r*= -0.336  *p*= 0.262 | *r*= -0.026  *p*= 0.933 |
|  |  |  |  |  |  |  |  |  |  |
| △ IED Completed Stages |  | *r*= -0.162  *p*= 0.598 | *r*= 0.063  *p*= 0.838 | *r*= -0.310  *p*= 0.303 | *r*= -0.309  *p*= 0.304 | *r*= 0.248  *p*= 0.413 | *r*= -0.197  *p*= 0.518 | *r*= 0.076  *p*= 0.804 | *r*= 0.050  *p*= 0.870 |
|  |  |  |  |  |  |  |  |  |  |
| △ IED Total Errors |  |  | *r*= 0.252  *p*= 0.406 | *r*= 0.060  *p*= 0.846 | *r*= 0.194  *p*= 0.526 | *r*= -0.310  *p*= 0.303 | *r*= -0.631  *p*= 0.021* | *r*= -0.551  *p*= 0.051 | *r*= -0.100  *p*= 0.746 |
|  |  |  |  |  |  |  |  |  |  |
| △ Outside-fMRI Error_sw_ (%) |  |  |  | *r*= -0.084  *p*= 0.785 | *r*= 0.165  *p*= 0.590 | *r*= -0.690  *p*= 0.009* | *r*= -0.046  *p*= 0.882 | *r*= 0.014  *p*= 0.965 | *r*= 0.140  *p*= 0.648 |
|  |  |  |  |  |  |  |  |  |  |
| △ Inside- fMRI Error_sw_ (%) |  |  |  |  | *r*= 0.519  *p*= 0.069 | *r*= -0.156  *p*= 0.610 | *r*= 0.062  *p*= 0.839 | *r*= 0.199  *p*= 0.514 | *r*= 0.059  *p*= 0.847 |
|  |  |  |  |  |  |  |  |  |  |
| △ Inside- fMRI RT_sw_ (ms) |  |  |  |  |  | *r*= -0.487  *p*= 0.091 | *r*= 0.105  *p*= 0.733 | *r*= -0.032  *p*= 0.917 | *r*= 0.043  *p*= 0.890 |
|  |  |  |  |  |  |  |  |  |  |
| △ Frequency of social interaction |  |  |  |  |  |  | *r*= -0.262  *p*= 0.387 | *r*= 0.134  *p*= 0.662 | *r*= -0.053  *p*= 0.863 |
|  |  |  |  |  |  |  |  |  |  |
| △BOLD in L SFG |  |  |  |  |  |  |  | *r=* 0.389  *p*= 0.152 | *r=* 0.224  *p*= 0.462 |
|  |  |  |  |  |  |  |  |  |  |
| △ BOLD in R MFG |  |  |  |  |  |  |  |  | *r=* 0.361  *p*= 0.225 |

Partial correlation analyses were performed, controlling for age, gender, and education. △= post-intervention value – pre-intervention value; BOLD= blood oxygenation level dependent; Error= error rate; IED= Intra-Extra Dimensional Set Shift; L SFG= left superior frontal gyrus; R MFG= right middle frontal gyrus; L IFG_t_= left inferior frontal gyrus pars triangularis; RT= reaction time; sw= Switch condition. *Significant correlations, *p* < 0.05.
