# Supplementary material for: Task-Switching Performance Improvements After Tai Chi Chuan Training Are Associated With Greater Prefrontal Activation in Older Adults
Source: Front Aging Neurosci. 2018 Sep 24;10:280. doi: 10.3389/fnagi.2018.00280 (PMC6165861; doi:10.3389/fnagi.2018.00280)
Supplement: Supplementary file 7 [file Table_4.docx]

**Supplementary Table S4. Inter-relationships among changes in task-switching performance, changes in physical function and social interaction, and changes in BOLD response magnitude in the prefrontal cortex during the Switch condition from pre-intervention to post-intervention in the CON group**

|  | △ IED Completed Stages | △ IED  Total Errors | △ Outside- fMRI Error_sw_ (%) | △ Inside- fMRI Error_sw_ (%) | △ Inside- fMRI RT_sw_ (ms) | △ Frequency of social interaction | △ BOLD  in L SFG | △ BOLD  in R MFG | △ BOLD  in L IFG_t_ |
| --- | --- | --- | --- | --- | --- | --- | --- | --- | --- |
| △ Knee extensor strength (kg) | *r*= -0.502  *p*= 0.204 | *r*= -0.413  *p*= 0.309 | *r*= -0.518  *p*= 0.188 | *r*= 0.903  *p*= 0.002* | *r*= -0.112  *p*= 0.792 | *r*= 0.100  *p*= 0.814 | *r*= -0.184  *p*= 0.662 | *r*= 0.058  *p*= 0.891 | *r*= 0.329  *p*= 0.426 |
|  |  |  |  |  |  |  |  |  |  |
| △ Four Square Step Test (sec) | *r*= 0.594  *p*= 0.121 | *r*= 0.343  *p*= 0.406 | *r*= 0.040  *p*= 0.926 | *r*= -0.098  *p*= 0.818 | *r*= 0.045  *p*= 0.916 | *r*= -0.363  *p*= 0.377 | *r*= -0.217  *p*= 0.606 | *r*= 0.306  *p*= 0.461 | *r*= -0.501  *p*= 0.206 |
|  |  |  |  |  |  |  |  |  |  |
| △ Six Minute Walk Test (m) | *r*= -0.048  *p*= 0.911 | *r*= 0.248  *p*= 0.553 | *r*= -0.396  *p*= 0.332 | *r*= 0.495  *p*= 0.213 | *r*= -0.075  *p*= 0.860 | *r*= 0.495  *p*= 0.213 | *r*= 0.495  *p*= 0.212 | *r*= 0.200  *p*= 0.634 | *r*= 0.221  *p*= 0.599 |
|  |  |  |  |  |  |  |  |  |  |
| △ IED Completed Stages |  | *r*= 0.782  *p*= 0.022* | *r*= 0.258  *p*= 0.538 | *r*= -0.340  *p*= 0.409 | *r*= 0.213  *p*= 0.613 | *r*= 0.012  *p*= 0.977 | *r*= 0.202  *p*= 0.632 | *r*= 0.238  *p*= 0.570 | *r*= -0.043  *p*= 0.920 |
|  |  |  |  |  |  |  |  |  |  |
| △ IED Total Errors |  |  | *r*= 0.203  *p*= 0.629 | *r*= -0.341  *p*= 0.409 | *r*= 0.076  *p*= 0.857 | *r*= -0.045  *p*= 0.916 | *r*= 0.509  *p*= 0.198 | *r*= 0.231  *p*= 0.581 | *r*= 0.241  *p*= 0.565 |
|  |  |  |  |  |  |  |  |  |  |
| △ Outside-fMRI Error_sw_ (%) |  |  |  | *r*= -0.382  *p*= 0.350 | *r*= 0.848  *p*= 0.008* | *r*= -0.423  *p*= 0.296 | *r*= 0.052  *p*= 0.903 | *r*= 0.138  *p*= 0.745 | *r*= -0.019  *p*= 0.965 |
|  |  |  |  |  |  |  |  |  |  |
| △ Inside- fMRI Error_sw_ (%) |  |  |  |  | *r*= 0.119  *p*= 0.778 | *r*= -0.017  *p*= 0.968 | *r*= -0.292  *p*= 0.483 | *r*= 0.150  *p*= 0.722 | *r*= 0.076  *p*= 0.858 |
|  |  |  |  |  |  |  |  |  |  |
| △ Inside- fMRI RT_sw_ (ms) |  |  |  |  |  | *r*= -0.298  *p*= 0.473 | *r*= -0.032  *p*= 0.940 | *r*= 0.256  *p*= 0.540 | *r*= -0.009  *p*= 0.983 |
|  |  |  |  |  |  |  |  |  |  |
| △ Frequency of social interaction |  |  |  |  |  |  | *r*= 0.434  *p*= 0.282 | *r*= -0.213  *p*= 0.612 | *r*= 0.107  *p*= 0.801 |
|  |  |  |  |  |  |  |  |  |  |
| △BOLD in L SFG |  |  |  |  |  |  |  | *r*= 0.496  *p*= 0.211 | *r*= 0.694  *p*= 0.056 |
|  |  |  |  |  |  |  |  |  |  |
| △ BOLD in R MFG |  |  |  |  |  |  |  |  | *r*= 0.564  *p*= 0.145 |

Partial correlation analyses were performed, controlling for age, gender, and education. △= post-intervention value – pre-intervention value; BOLD= blood oxygenation level dependent; Error= error rate; IED= Intra-Extra Dimensional Set Shift; L SFG= left superior frontal gyrus; R MFG= right middle frontal gyrus; L IFG_t_= left inferior frontal gyrus pars triangularis; RT= reaction time; sw= Switch condition. *Significant correlations, *p* < 0.05.
